# Regulation of Stress Responses and Translational Control by Coronavirus

**DOI:** 10.3390/v8070184

**Published:** 2016-07-04

**Authors:** To Sing Fung, Ying Liao, Ding Xiang Liu

**Affiliations:** 1 School of Biological Sciences, Nanyang Technological University, 60 Nanyang Drive, Singapore 637551, Singapore; tosingfung@163.com; 2 Department of Avian Diseases, Shanghai Veterinary Research Institute, Chinese Academy of Agricultural Sciences, Ziyue Road 518, Shanghai 200241, China

**Keywords:** coronavirus, ER stress, unfolded protein response, p38, JNK, eIF2α, PKR, PERK, GADD34/PP1, nsp1, translational control

## Abstract

Similar to other viruses, coronavirus infection triggers cellular stress responses in infected host cells. The close association of coronavirus replication with the endoplasmic reticulum (ER) results in the ER stress responses, which impose a challenge to the viruses. Viruses, in turn, have come up with various mechanisms to block or subvert these responses. One of the ER stress responses is inhibition of the global protein synthesis to reduce the amount of unfolded proteins inside the ER lumen. Viruses have evolved the capacity to overcome the protein translation shutoff to ensure viral protein production. Here, we review the strategies exploited by coronavirus to modulate cellular stress response pathways. The involvement of coronavirus-induced stress responses and translational control in viral pathogenesis will also be briefly discussed.

## 1. ER Stress Responses Regulated by Coronavirus and Its Implication in Pathogenesis

### 1.1. The Integrated Signaling Network of the Unfolded Protein Response (UPR)

**Figure 1:**
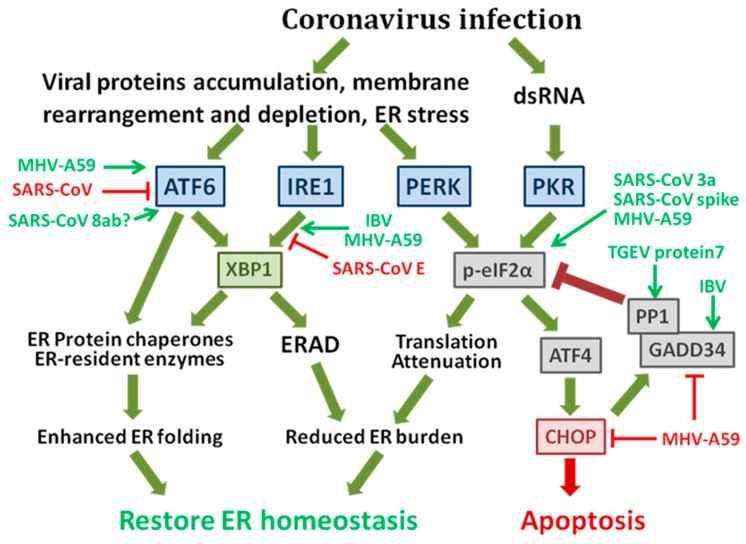
The induction of ER stress and UPR during coronavirus infection. Coronavirus infection induces ER stress and activates UPR. Activated ATF6 transcriptionally induces XBP1, ER chaperones and enzymes to enhance the ER folding capacity. Activated IRE1 mediates splicing of the XBP1 mRNA, whereas the spliced XBP1 protein enhances ER folding and reduces ER burden by promoting ERAD. Activated PERK mediates the phosphorylation of eIF2α, leading to a global translation attenuation. Signaling via the ATF4-CHOP pathway promotes apoptosis induction during prolonged ER stress. The known coronaviruses and viral proteins modulating the UPR signaling are also indicated. Green arrows are activating and red blunt arrows are inhibiting. See text for detail.

Inside a eukaryotic cell, most of the transmembrane and secreted proteins are translated, modified, and folded in the ER. The amount of proteins in the ER can fluctuate substantially when a cell is undergoing physiological changes or when it is affected by various environmental stimulations. If the protein influx overloads the protein processing machinery, unfolded/misfolded proteins will accumulate inside the ER and result in ER stress. In order to return to homeostatis, cells have evolved UPR [1], which is composed of three pathways. These pathways are initiated by three ER sensor proteins located in the ER: PKR-like ER protein kinase [1], activating transcriptional factor-6 (ATF6) and inositol-requiring protein-1 (IRE1) (Figure 1). All of them are single-pass transmembrane proteins, consisting of a luminal domain that recognizes unfolded/misfolded protein inside the ER, and a cytosolic domain that ultimately relays the signal to the nucleus and switches on a specific set of downstream genes.

Under ER stress, oligomerization and activation PERK mediates the phosphorylation of eIF2α, resulting in the shutdown of global translation [2,3]. The PERK-dependent inhibition of protein synthesis limits nascent protein transport to ER lumen, thereby attenuating the protein accumulation in ER. Interestingly, some proteins are preferetially translated when eIF2α is phosphorylated. One example is ATF4 [4], a transcription factor that control the expression of genes involved in amino acid metabolism and transport and redox chemistry. GADD34 is one of downstream genes triggered by ATF4. As a regulator subunit, GADD34 helps PP1 to dephosphorylate eIF2α, thereby limiting PERK signaling as a negative feedback loop [5]. PERK signaling will be considered as part of translational control and will be discussed in Section 3.

As for the IRE1 branch of UPR, activation of IRE1 by auto-phosphorylation activates its cytosolic RNase domain, which mediates a unique splicing event that removes an intron from the transcript of X-box protein 1 (XBP1) [6]. The spliced form of XBP1 protein (XBP1s) is then translated and imported to the cell nucleus, thereby activating the expression of UPR genes, which encode various ER protein chaperones as well as components of the ER-associated degradation (ERAD) pathway [6]. Moreover, IRE1 is also known to catalyze non-specific degradation of mRNAs associated with the ER, a phenomenon dubbed as IRE1-dependent RNA decay (RIDD) that effectively reduces the translational burden of the ER [7]. In spite of these pro-survival activities, prolonged activation of IRE1 can also activate c-Jun N-terminal kinase (JNK) and promote caspase-12 dependent apoptosis [8,9].

In terms of ATF6, increasing amounts of unfolded proteins activate the protein and lead to its translocation from the ER to the Golgi, in which the protein is sequentially cleaved by proteases. The cytosolic domain of ATF6 is then released and transported into the nucleus[10], where it induces the expression of UPR genes, such as some ER protein chaperones (calreticulin, glucose regulated protein 78 kDa (GRP78) and GRP94), some ERAD proteins, as well as ER-resident enzymes (protein disulfide isomerase) [11].

With the help of several feedback mechanisms, the three UPR pathways mentioned above actually constitute an inter-related signaling network [1]. For example, XBP1 mRNA from the IRE1 branch has been shown to be induced by PERK and ATF6 when cells are under ER stress [6,12]. Moreover, both PERK and PKR could be inhibited by P58^IPK^, which is a downstream gene transcriptionally induced by XBP1s [13,14]. Finally, the expression and activation of ATF6 could be enhanced by PERK, while ATF6 may induce protein disulfide isomerase A6, which promotes the degradation of IRE1 [15,16,17]. Hence, the three branches of UPR should be recognized as a closely interrelated and intricately regulated signaling network.

### 1.2. The Possible Mechanisms of Coronavirus-Induced ER Stress Responses

Increased expression of GRP78, GRP94 and other ER stress related genes has been determined in cells infected with various coronaviruses. These include mouse hepatitis virus (MHV), severe acute respiratory syndrome coronavirus (SARS-coronavirus) and infectious bronchitis virus (IBV) [18,19,20]. Activation of ER stress response can be also detected in cell overexpressing the SARS-coronavirus spike protein [21,22], protein 3a [23], protein 6 [24], protein 8a, and protein 8b [25].

Although not completely illustrated, it is proposed that coronavirus induces ER stress via three potential processes [26]. First, translation, folding and modification of large amounts of coronavirus structural proteins, in particular the heavily N-link glycosylated spike protein, significantly increases the burden of the ER in infected cells. Indeed, overexpression of the spike protein from SARS-coronavirus [21], MHV [20] or IBV can all activate UPR. Maturation of the spike protein could further exhaust chaperones inside the ER lumen such as calnexin, which is known to physically bind to the SARS-coronavirus spike protein to facilitate its proper folding [27]. Second, in order to assemble the replication/transcription complex, coronavirus induces the rearrangement of ER membrane into double membrane vesicles (DMVs), zippered ER or ER spherules [28,29]. DMVs formation could be observed in cells overexpressing the SARS-coronavirus non-structural protein nsp3, nsp4 and nsp6, suggesting their involvement in coronavirus-induced membrane remodeling [30]. Moreover, DMVs formed in cells infected with SARS-coronavirus are derived from a modified reticulovesicular network connecting to the ER, as determined by high resolution electron tomography [31]. Also, MHV may hijack vesicles originated from the ER and use the membrane for DMV formation [32]. Finally, morphogenesis and budding of mature virus particles deplete the membrane component in the ER. Assembly and budding of coronavirus virions occur in the ER-Golgi intermediate compartment (ERGIC), a membrane system that is structurally and functionally extended from the ER [33,34]. It has been well established that depletion of ER lipid components (such as phosphatidylcholine) alters the morphology of ER and pertubs trafficking of protein cargo in the Golgi [35].

Taken together, current evidence demonstrates that coronavirus infection induces ER stress and triggers UPR in general. It is apparent that coronavirus might subvert or utilize certain aspects of the UPR to benefit its own replication and pathogenesis.

### 1.3. Coronavirus-Induced UPR and Its Implication in Pathogenesis

The IRE1-XBP1 pathway is activated by MHV [36] and IBV infection [37], which may be caused by the accumulation of the spike protein in ER lumen [20]. However, either SARS-coronavirus infection or SARS-coronavirus spike protein overexpression does not lead to XBP1 splicing [20], suggesting the modulation of UPR branches differs for different coronaviruses. Interestingly, when the E gene is deleted from the SARS-coronavirus genome, the resulting mutant virus (rSARS-coronavirus-ΔE) is significantly attenuated, but significantly activates XBP1 splicingand up-regulates other cellular stress genes, leading to increased apoptosis [38]. The study suggests that SARS-coronavirus E protein suppresses the IRE1-XBP1 pathway activation and inhibits apoptosis induction, although the mechanism remains unknown.

As for MHV, in spite of the observed XBP1 mRNA splicing, neither the spliced XBP1 protein (XBP1s) nor the upregulation of downstream UPR genes could be detected, presumably resulted from the sustained translational suppression due to eIF2α phosphorylation [36]. Conversely, significant upregulation of UPR genes (such as ERdj4 and p58^IPK^) can be detected in cells infected with IBV, indicating full activation of the IRE1-XBP1 pathway [37]. Moreover, RNA interference of IRE1 and inhibition of XBP1s drastically potentiate IBV-induced apoptosis, whereas overexpression of IRE1 and XBP1s promotes cell survival, pointing to an anti-apoptotic nature of the IRE1-XBP1 pathway during IBV infection [37]. Activation of a pro-survival response at the late stage of infection could benefit the virus, by giving more time for virus particle assembly and release before the infected cells are disintegrated. However, knockdown of IRE1 does not significantly affect the production of infectious IBV in the supernatant [37]. Therefore, the physiological significance of IRE1 during coronavirus infection needs to be determined with further experiments and with appropriate in vivo models.

Apart from apoptosis, activation of IRE1 may affect other cellular events that modulate the pathogenesis of coronavirus infection. For example, IRE1 can also modulate pro-inflammatory response by activation of nuclear factor kappa-light-chain-enhancer of activated B cells (NF-κB) and induction of cytokines such as interleukin 6 (IL-6) and IL-8 [39,40]. On the other hand, XBP1 has been demonstrated to directly or indirectly modulate the expression of various cytokines and type-I interferons (IFNs) [41,42,43]. Unpublished data from this group have also implicated the involvement of IRE1 and XBP1 in the transcription of IL-8 and IFN-βduring IBV infection. Further investigation needs to be carried out for other coronaviruses to understand the detailed mechanisms and potential counter measures implemented by coronaviruses.

Whether or not coronavirus activates the ATF6 branch of UPR during coronavirus infection has not been fully investigated. Proteolytic cleavage of ATF6 into its active form cannot be detected in cells infected with SARS-coronavirus [38]. Similarly, ATF6 reporter constructs co-transfected with the SARS-coronavirus spike protein showed minimal reporter production [21]. In contrast, ATF6 cleavage is observed by MHV infection, although both the full-length and the cleaved form of ATF6 decreased significantly at late stage of infection due to sustained translational attenuation [36]. Subsequently, neither the induction of downstream UPR genes nor the activation of the ERSE reporter construct could be detected. Interestingly, the SARS-coronavirus 8ab protein binds to ATF6 in vitro and promotes its cleavage in the co-transfected cells [25]. However, further experiments using recombinant viruses are required.

Coronavirus also manipulate PERK activity and the level of phosphor-eIF2α to control protein synthesis, such as IBV [18,44], MHV [45], SARS-coronavirus [23,46,47]. The regulation of PERK-eIF2α pathway by various coronaviruses will be discussed in detail in Section 3. The regulation of ER stress response pathways by coronavirus is summarized in Figure 1.

## 2. Activation and subversion of p38 and JNK signaling pathways by coronavirus infection

### 2.1. The Signaling Pathways of Stress-Activated Protein Kinase p38 and JNK

The mitogen activated (MAP) kinases are evolutionarily conserved protein kinases that regulate a diversity of critical cellular signaling pathways, such as cell division, differentiation, autophagy, apoptosis, innate immunity and pro-inflammatory response [48]. So far, four subgroups of MAP kinases have been identified in metazoans, namely the extracellular regulated kinase 1/2 (ERK1/2), ERK5, the p38 MAP kinase and the c-Jun N-terminal kinases (JNK) [49,50]. Among them, the ERK1/2 signaling is triggered by growth factors and mitogens, whereas the p38 and JNK are mainly responsive to cellular stress, such as DNA damage, ionizing radiation and protein synthesis inhibition [49].

MAP kinases are phosphorylated by the upstream MAP kinase kinases (MAPKKs), which are in turn phosphorylated by upstream kinases in response to different cellular or environmental stimulations [51]. In particular, p38 is activated by MKK3, MKK4 [52] or MKK6 [53], whereas JNK can be phosphorylated by MKK4 or MKK7 [54]. Dual phosphorylation of Thr and Tyr residues in the conserved TxY motif (Thr-Gly-Tyr for p38 and Thr-Pro-Tyr for JNK) is essential for the complete activation of MAP kinases [49].

Upon activation, p38 translocates into the nucleus and activates multiple effector proteins, such as MAPK-Activated Protein Kinase-2 (MAPKAPK2), which in turn activates critical transcription factors such as cAMP Response Element-Binding protein (CREB) and Activating Transcription Factor 1 (ATF1) [55]. Moreover, p38 can also directly phosphorylate other important transcription factors such as p53 and CHOP [56,57].

As for JNK, activated JNK phosphorylates c-Jun and other downstream substrates, enhancing their transcription activity [58]. Activated c-Jun dimerizes with other proteins such as cellular FBJ murine osteosarcoma (c-Fos) to form the activator protein 1 complex, thereby inducing genes harboring the 12-*O*-tetradecanoylphorbol-13-acetate (TPA) response element [59].

### 2.2. Induction and Subversion of the p38 Pathway by Coronavirus Infection

Phosphorylation of p38 is detected in cells infected with several coronaviruses including MHV [60], SARS-coronavirus [61], feline coronavirus (FCoV) [62], IBV [63] and transmissible gastroenteritis coronavirus (TGEV) [64]. Moreover, the p38 pathway is also activated in cells overexpressing the SARS-coronavirus accessory protein 3a [65] and 7a [66], although the physiological significance during an actual infection has not been fully examined. Coronavirus-induced p38 activation is likely mediated by the upstream kinase MKK3/6, which is phosphorylated in cells infected with MHV [67] and IBV [63].

Multiple reports have suggested the critical role of p38 in the inflammatory process during coronavirus infection. For example, inhibition of p38 significantly reduces the transcription and secretion of IL-6 in MHV-infected cells [67]. Similarly, the induction of IL-6 and IL-8 is dependent on p38 activation in IBV-infected cells [63]. Moreover, production of tumor necrosis factor alpha (TNF-α) and IL-1β in FIPV-infected cells is significantly inhibited by p38 inhibitor [62]. Finally, p38 has been demonstrated to induce the expression of macrophage prothrombinase fibrinogen-like protein 2 (Fgl2), a protein crucial for the pathogenesis of fulminant hepatitis in MHV-3-infected mice [60,68].

Notably, coronaviruses have developed various mechanisms to subvert the activation of the p38 pathway. For instance, IBV has been shown to induce the transcription of dual-specificity phosphatase 1 (DUSP1), which can dephosphorylate the active p38 and suppress the production of excessive pro-inflammatory cytokines [63]. In contrast, the SARS-coronavirus E protein seems to activate p38 and induces inflammation [69]. Specifically, the PDZ-binding motif (PBM) in the SARS-coronavirus E protein can interact with the cellular protein syntenin, resulting in its redistribution from the nucleus to the cytoplasm and activation of the p38 pathway [69]. In fact, production of inflammatory cytokines is significantly reduced in syntenin-knockdown cells, or in mice infected with PBM-deleted E recombinant virus compared with mice infected with the wild type virus [69]. Therefore, different coronaviruses may use distinct mechanisms to modulate the p38 pathway, so as to establish the cellular environment suitable for viral replication and spreading.

### 2.3. Activation of JNK during Coronavirus Infection

Compared with p38, coronavirus-induced activation of the JNK pathway has been less well characterized. JNK phosphorylation has been determined in cells infected with MHV [67], SARS-coronavirus [67] and IBV [37]. Activation of the JNK pathway has also been demonstrated in cells overexpressing the N protein, accessory protein 3a, 3b or 7a of SARS-coronavirus [70,71,72].

In a study using specific inhibitors, Mizutani et al have shown that persistent SARS-coronavirus infection requires the intact signaling of JNK and Akt in Vero E6 cells, suggesting a pro-survival function for these kinases [73]. Phosphorylation of JNK and Akt is likely induced by the SARS-coronavirus N protein, and the anti-apoptotic Bcl2 and Bcl-xL proteins may also contribute to the persistent infection [74]. In contrast, JNK signaling has been found to facilitate apoptotic cell death in IBV-infected cells [37]. Therefore, it is possible that JNK serves different functions in cells infected with different coronaviruses, and presumably at different stages of infection.

In terms of its involvement in proinflammatory response, induction of TNF-α and IL-6 by MHV-A59 infection in primary mouse astrocytes depends on JNK, but not NF-κB or other MAP kinases [75]. The SARS-coronavirus spike protein also activates the protein kinase C epsilon, which induces JNK phosphorylation in a calcium-independent manner. Moreover, induction of IL-8, activation of CREB and the transcription of cyclooxygenase-2 (COX-2) gene in cells transfected with SARS-coronavirus spike also requires JNK [76,77].

**Figure 2:**
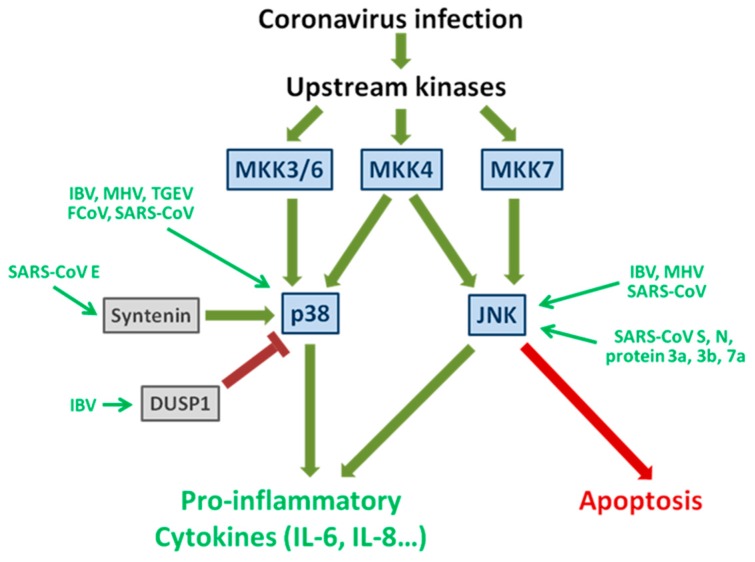
Activation and subversion of p38 and JNK signaling pathways by coronavirus infection. Coronavirus infection activates upstream kinases, which in turns activate MKK3/6, MKK4 and MKK7. JNK is phosphorylated by MKK4 and/or MKK7, while p38 is activated by MKK3/6 and/or MKK4. Both p38 and JNK have been shown to modulate inflammatory response by regulating the production of pro-inflammatory cytokines (such as IL-6, IL-8 and TNF-α). JNK has also been implicated in the induction of apoptosis in coronavirus-infected cells. The known coronaviruses and viral proteins modulating p38 and JNK signaling are also indicated. Green arrows are activating and red blunt arrows are inhibiting. See text for detail.

The modulation of MAPK pathways by coronavirus is summarized in Figure 2.

## 3. Translational Control by Coronavirus and Its Implication in Pathogenesis

### 3.1. Viral Protein Translation of Coronavirus

Coronavirus replicate entirely within the cytoplasm of their host cells, where they produced five to nine genomic mRNAs [41]. All mRNAs contain a common 5′-leader sequence (65–90 nucleotides long) and a co-terminal 3′-end [78]. The 5′-leader sequence binds to N protein and form a complex, may act as a strong translation initiation signal and promote viral mRNA translation [79,80]. For each mRNA, only the 5′-open reading frames (ORFs) are translated via cap-dependent manner. Most of the mRNAs are monocistronic, while some mRNAs are bicistronic or tricistronic. mRNA1 encodes two large precursor polyproteins pp1a and pp1ab, pp1ab is translated via −1 programmed ribosomal shifting manner. Both polyproteins pp1a and pp1ab are proteolytically cleaved by virus encoded proteases, papain-like proteinase (PL^pro^) and 3C-like proteinase (3CL^pro^), into 13–16 mature non-structural proteins (nsps). Many of the nsps participate in viral RNA replication and transcription. The subgenomic mRNAs are translated into structural proteins haemagglutinin-esterase (HE), spike protein (S), envelope protein (E), membrane protein (M), nucleocapsid protein (N), and several nsps, respectively. HE is only encoded by β coronavirus. The expression ratio of these genes is regulated at the level of transcription, during which time the shorter mRNAs are produced more abundantly than the longer one [81,82]. The 5′-UTR (untranslated region), which is unique for each of mRNA, also regulate the rate of each mRNA translation [83].

Viruses utilize host translation machinery to finish viral protein translation. In response to acute viral infection, host cell would shut down protein translation system to cope with the infection stress, which is regarded as integrated stress response. Integrated stress response is marked by phosphorylation of eIF2α, down regulation of the general cap-dependent protein synthesis, and up-regulation of the expression of certain transcription factors, such as ATF4. To acquire successful production of viral proteins, viruses must overcome this obstacle to ensure viral protein synthesis. The translation regulation by virus infection occurs by a number of ways, such as degradation of cellular mRNAs, alteration the activity of ribosome and associated factors, competitive displacement of cellular mRNAs by viral mRNAs for translation, etc. Coronavirus mRNAs are 5′-capped and 3′-polyadenylated, structurally equivalent to host mRNAs. Due to the translation competition between cellular and viral mRNAs for limiting number of ribosomes and associated factors, coronavirus must hijack the host translational machinery to produce their own proteins. More and more studies focus the mechanisms of translational control by coronavirus. SARS-coronavirus [84,85,86,87], MERS-coronavirus [88], MHV [36,89,90], transmissible gastroenteritis virus (TEGV) [91,92], porcine epidemic diarrhea virus (PEDV) [93], bat coronaviruses [94], have been shown to induce host translation shutoff in susceptible cells. In the other way, infectious bronchitis virus (IBV) maintains protein translation in infected cells [44]. The mechanisms of coronavirus modulated translation will be summarized in following sections.

### 3.2. Regulation of Host Protein Synthesis via Targeting to eIF2α

Host protein translation shutoff is not only due to the host mRNA degradation and specific viral protein induced ribosome disfunction, but also induced by virus infection stress stimuli [95]. The infection stress stimili induces translational shut off via the phosphorylation of eukaryotic initiation factor α (eIF2α). eIF2α, together with eIF2β, and eIF2γ, forms eIF2, mediates the binding of initiator Met-tRNAi to the ribosome in a GTP-dependent manner. Once the initiation is completed, eIF2-GDP is released from the ribosome, and GDP is exchanged for GTP to form active eIF2-GTP, participating in another round of translation initiation. eIF2 is inactivated by phosphorylation of eIF2α on Ser51, which has increased affinity to eIF2β and blocks the exchange of GDP to GTP, thus depleting the active eIF2β-GTP pool. Since the cellular concentration of eIF2β is much lower than eIF2α, a small proportion of phosphor-eIF2α can exhaust eIF2β by sequestration [96]. Therefore, phosphorylation of eIF2α is a checkpoint of protein synthesis initiation and relieves stress through ceasing translation under infection stress. Given the importance of eIF2α in translation initiation, the level of phosphor-eIF2α is no wonder regulated under infection stress. There are four eIF2α kinases, GCN2, HRI, PERK, and PKR. These kinases are activated as a result of discrete stress, such as amino acid starvation or ultraviolet light (GCN2), heme deficiency (HRI), excess unfolded proteins accumulated in the ER [97], and double-stranded RNA (dsRNA) produced in virus-infected cells (PKR) [98].

Under virus infection stress, PKR is induced as an IFN stimulated gene (ISG) and activated by dsRNA produced during the course of viral infection. dsRNA binds to PKR and causes a conformational change, leading to the dimerization and autophosphorylation of this kinase [99]. PKR phosphorylates eIF2α and inhibits protein synthesis, thereby rendering an antiviral effect [96]. PERK, another eIF2α kinase, usually activated by excess viral proteins loaded in the ER [2,100]. PKR and PERK activation inhibits protein synthesis globally and curtails viral spread through inactivating eIF2α making these two kinases key players in integrated stress response to virus infection. High level of phosphor-eIF2α leads to preferential translation of transcription factor ATF4, which promotes a number of genes expression. One of ATF4 target genes is growth arrest and DNA damage-inducible protein 34 (GADD34), a regulatory subunit of protein phosphatase 1 (PP1). GADD34 helps PP1 to dephosphorylate eIF2α, thereby recovering global protein synthesis. The induction of GADD34 is a canonical cellular response to counteract PERK/PKR via preserving eIF2α activity [5,101]. A number of viruses encode viral protein mimicing the function of GADD34, helps PP1 to counterbalance the PKR/PERK-mediated phosphorylation of eIF2α.

Regarding to coronavirus, it was reported that SARS-coronavirus infection activates PERK by phosphorylation, leading to sustained phosphorylation of eIF2α in 293T/ACE2 cells. The SARS-coronavirus infection also activates PKR. Unexpectedly, the activation of PKR is not involved in eIF2α phosphorylation and virus replication, but plays a role in viral induced apoptosis [47]. Overexpresson of SARS-coronavirus spike glycoprotein resulted in activation of PERK and upregulation of GRP78/GRP94 [46]. Another ER localized transmembrane glycoprotein, 3a, also activates PERK and thus increases the level of phosphor-eIF2α, leading to activation of GRP78, GRP94, ATF4, and CHOP promoter, which is demonstrated by co-transfection of luciferase reporter plasmid with plasmid encoding 3a [23]. It is unclear whether spike protein or 3a protein plays a major role in SARS-coronavirus induced translation shutoff, as the translational rate is not examined upon overexpression of these proteins.

MHV strain A59 infection increases the level of phosphor-eIF2α and attenuates the host protein synthesis, resulting in preferential translation of ATF4. Unexpectedly, the ATF4 target genes, GADD34 and CHOP, are not detected during MHV infection, which may correlates with the persistent phosphorylation of eIF2α and sustained suppression of host protein synthesis [36,45]. The MHV induced translation inhibition coincides with degradation of host mRNA. The formation of stress granules and P bodies, the sites of mRNA stalling and degradation respectively, are detected in MHV-infected LR7 cells [102]. MHV infection also leads to RNase L-independent specific 28S rRNA cleavage in all susceptible cell lines, which may contribute to MHV induced translational inhibition [103]. Although MHV infection induces protein synthesis inhibition, the viral proteins are still efficiently made, which can be attributed to the 5′-leader sequence and N protein in viral mRNA or the abundance of viral mRNA. MHV replication is not negatively affected either in the host translational shutoff-deficient (eIF2αS51A) MEF cells or stress granule impaired (TIA-1^−/−^ and TIAR^−/−^) MEFs, suggesting the translational inhibition is not beneficial for virus replication in vitro [102].

To allow adequate proteins synthesis, some coronaviruses harness strategies to prevent eIF2α phosphorylation. For example, IBV activates PKR and PERK at early infection times, leading to phosphorylation of eIF2α, however, the activation of PKR and PERK is suppressed at late infection times by unknown mechanisms Furthermore, IBV infection up-regulates GADD34 expression, which is a regulatory subunit of PP1. By suppression of kinase PERK and PKR activity, and enhancement of phosphotase GADD34-PP1 activity, phosphor-eIF2α was reduced to low level at late infection times, resulting in robust protein synthesis throughout the infection course. The sustained protein translation is beneficial for virus replication, which is verified by overexpression of GADD34 [18,44]. Unlike IBV, which maintains protein synthesis via blockage of PKR/PERK activity and up-regulation of GADD34, TEGV infection leads to PKR activation with a maximum at 12 h.p.i.. However, the level of phosphor-eIF2α in TEGV infected cell is comparable to mock-infected cells, and moderate protein synthesis is observed throughout the infection times. A genus-specific protein 7 mimicking the function of GADD34 is identified in TEGV, with a conserved sequence motif (RVIFLVL) binding to PP1c. The interaction of protein 7 and pp1c is required for dephosphorylation of eIF2α, inhibition of cellular RNA degradation, and maintenance of protein synthesis. Infect cells with TGEVΔ7 (with deletion of protein 7) results in impaired protein synthesis, faster and stronger 28S rRNA degradation, and increased apoptosis, compared to that in wild type TEGV infected cells. Moreover, TGEVΔ7 infected piglets exhibits accelerated pathology and more lesions at 4 dpi when compared with the wild type TGEV infected one. Piglets infected with virulent enteric strain TGEV-SC11-Δ7 develop faster and more pronounced clinical disease. Thus, TGEV protein 7 functions to relieve the integrated stress response and to recover protein synthesis, thereby attenuating virus virulence [104].

### 3.3.Suppression of Host Protein Synthesis and IFN Response by Coronavirus nsp1

Coronavirus nsp1, the most N-terminal product of the pp1a polyprotein, is only encoded by two genera, α and β coronavirus. This protein is highly divergent among coronaviruses and functions to regulate host and viral gene expression. Recently, a number of reports demonstratethat nsp1 is involved in the regulation of host protein translation. Among β coronavirus, SARS-coronavirus nsp1 was first identified to block the expression of reporter gene under the control constitutive promoters [84]. This protein associates and inactivates the 40S ribosomal subunit, thus preventing viral and cellular mRNA translation [105]. Moreover, the Nsp1-40S ribosome complex also promotes cellular mRNA degradation via inducing an endonucleolytic mRNA cleavage in the 5′ region of the capped mRNA [84,105,106]. The presence of 5′-end leader sequence in viral mRNA, and the binding of stem loop 1 in the 5′-UTR with nsp1, protect viral mRNAs from nsp1-induced RNA cleavage, and facilitate efficient viral gene expression in infected cells [87,106]. In vivo studies suggested that SARS-coronavirus nsp1 counteracts the host innate immune response via suppression of host gene expression, including type I IFN, thereby playing a crucial role in virus virulence [85,105,107]. The inhibition of host protein synthesis and IFN inhibition by nsp1 seems to be a general phenomenon in coronavirus, which is also observed in other coronaviruses. For example, the nsp1 of bat coronavirus strains, Rm1, 133 and HKU9-1, exhibit functional similarities with SARS-coronavirus nsp1, with the ability to prevent host protein synthesis and suppress innate immune functions [94,108]. MERS-coronavirus nsp1 also negatively regulate host gene expression by inducing the degradation and inhibiting the translation of host mRNA. This protein selectively targets nuclear host mRNA and transports them to the cytoplasm for degradation and translation inhibition [88]. MHV nsp1 displays similar function to suppress the expression of reporter genes and host genes [108]. Deletion or mutation of potential functional domain of nsp1 severely attenuates MHV, and influences hepatotropism and liver pathogenesis in vivo, and elicits potent immune response. Thus, nsp1 is a major virulence factor [108,109,110].

Among α coronavirus, TEGV nsp1 suppresses host mRNAs translation without affecting their stability. This protein also inhibits the translation of different reporter mRNAs in cells or cell extract, but not in the in vitro translation system such as rabbit reticulocyte lysate, indicating that TEGV nsp1 relies on host factors to exert the inhibitory effect [91]. HCoV-229E and HCoV-NL63 nsp1 inhibit the expression of reporter genes, probably via binding to ribosomal protein S6 and blocking the mRNA binding to the 40S ribosomal subunit [108,111]. The essential role of covonavirus nsp1 in regulation of protein synthesis and IFN response, makes it a virulence factor and was targeted to developed live attenuated coronavirus vaccine [85,107,110].

## 4. Conclusions

Replication of coronavirus induces UPR and other cellular stress responses in the infected cells, triggering innate immunity and antiviral signaling pathways. The translational control constitutes one aspect of cellular antiviral response, and it is targeted and subverted by various coronaviruses at different levels via different mechanisms. It is hoped that further studies on these subjects will promote a more complete understanding of corovirus-host interactions and shed light on the development of antiviral therapeutics.
